# Ostracod Genetic Variability in Response to Lake Level‐Driven Salinity Dynamics in Nam Co, Central Tibetan Plateau, in the Past Two Millennia

**DOI:** 10.1002/ece3.72706

**Published:** 2025-12-26

**Authors:** Katharina Dulias, Sten Anslan, Luis Victoria Nogales, Anja Schwarz, Wengang Kang, Paula Echeverría‐Galindo, Nicole Börner, Ping Peng, Yongqin Liu, Keshao Liu, Bernd Wünnemann, Junbo Wang, Antje Schwalb

**Affiliations:** ^1^ Institute of Geosystems and Bioindication Technische Universität Braunschweig Braunschweig Germany; ^2^ Department of Biological and Environmental Science University of Jyväskylä Jyväskylä Finland; ^3^ Institute of Tibetan Plateau Research Chinese Academy of Sciences Beijing China; ^4^ Institute of Organic Biogeochemistry in Geo‐Systems (OBG) RWTH Aachen University Aachen Germany; ^5^ Faculty of Geosciences and Environmental Engineering Southwest Jiaotong University Chengdu China

**Keywords:** intra‐species diversity, metabarcoding, Nam Co, ostracods, sedaDNA, Tibetan Plateau

## Abstract

Sedimentary ancient DNA metabarcoding has revolutionized the reconstruction of past lake biodiversity, providing insights into species assemblages spanning thousands of years. We apply this approach to investigate ostracod community dynamics and genetic diversity in a two‐millennia sedimentary record from Nam Co, the third largest lake on the Tibetan Plateau, where hydrological fluctuations have driven shifts in salinity and habitat conditions. Using stratigraphic sedaDNA analyses, temporal diversity indices, and environmental proxies, we linked ostracod assemblages to lake‐level and climatic changes. Species richness peaked between 628 and 765 ce during moister conditions with increased minerogenic influx, and again since the mid‐1970s amid recent warming and glacier‐melt–driven lake‐level rise. In the earliest sedaDNA record (~230 bce), *Tonnacypris gyirongensis* indicated warm, nutrient‐rich, shallow estuarine conditions. Between ∼600 and ∼830 ce, *Candona candida* and *Ilyocypris angulata* reflect freshwater, organic‐rich littoral habitats. During the Medieval Climate Anomaly (~948–1143 ce) and Little Ice Age (~1600–1678 ce), alternating dominance of *Limnocythere* sp. and *Leucocytherella sinensis* primarily signals changes in water level and salinity due to their different ecological tolerances. Genetic data reveal generally low lineage diversity in parthenogenetic taxa, punctuated by transient increases in phylogenetic diversity during moister intervals at the end of the Late Antique Cold Period (490–830 ce), the late Medieval Climate Anomaly period (1250–1400 ce), and recent warming. Sediments spanning the Little Ice Age exhibit stable richness but shifts in dominance patterns, indicating a change in salinity and alkalinity. Since the 1950s, a marked turnover has occurred with declining *L. sinensis* and rising *Limnocythere inopinata* , coinciding with reduced monsoon runoff; in contrast, the post‐1970s freshening has promoted the resurgence of *L. sinensis* , alongside minor occurrences of *I. angulata* and *L. inopinata* . Our results demonstrate the sensitivity of Tibetan Plateau ostracod communities to hydrological and climatic variability. By integrating sedaDNA with paleoenvironmental proxies, we reveal both community‐level and intra‐species genetic responses to past climate change, underscoring the power of sedaDNA metabarcoding for paleoecological and evolutionary research.

## Graphical abstract

Using sedimentary ancient DNA metabarcoding on a two‐millennia sediment core from Nam Co (Tibetan Plateau), the study reconstructs ostracod community shifts and genetic diversity in response to hydrological and climatic changes. Species richness peaked during moister intervals, while alternating dominance during the Medieval Climate Anomaly and Little Ice Age reflected water‐level and salinity fluctuations; overall lineage diversity in parthenogenetic taxa was low, with transient phylogenetic boosts in wetter phases. These findings demonstrate the sensitivity of ostracod assemblages to past climate‐driven hydrology and underscore the power of sedaDNA metabarcoding for paleoecological and evolutionary research.

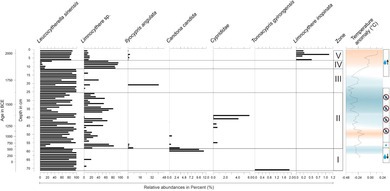

## 1 Introduction

Ostracods, a subclass of microcrustaceans, are integral to aquatic ecosystems and serve as valuable indicators in paleoenvironmental studies. On the Tibetan Plateau (TP), approximately one‐third of ostracod species are endemic, highlighting a significant rate of endemism (Wrozyna, Frenzel, Steeb, et al. 2009; Peng et al. 2021). Nam Co, situated at an elevation of ~4723 m a.s.l. in the central Tibetan Plateau, and one of the world's largest high‐altitude brackish lakes on the TP (Zhang et al. 2008; Keil et al. 2010; Wrozyna et al. 2010), has been the focus of extensive ostracod research (Wrozyna, Frenzel, Steeb, et al. 2009; Wrozyna, Frenzel, Xie, et al. 2009; Bonilla‐Flores et al. 2024). Studies have identified nine ostracod species across six genera from sediment cores and surface samples (Wrozyna, Frenzel, Steeb, et al. 2009; Wrozyna, Frenzel, Xie, et al. 2009; Zhu et al. 2010). The dominant species, *Leucocytherella sinensis*, is endemic to the Plateau and prevalent in Nam Co (Wrozyna et al. 2011). Ostracod assemblages and their stable isotope compositions have been utilized to reconstruct Holocene lake level fluctuations, offering insights into past monsoon variability and climatic conditions (Frenzel et al. 2010; Wrozyna et al. 2011), as well as Late Quaternary variations in the Asian monsoon system (Börner et al. 2022).

Over the past two millennia, Nam Co has experienced significant climatic fluctuations that have influenced its hydrology and ostracod assemblages (Xia et al. 2008; Mügler et al. 2010; Wrozyna et al. 2010; Kasper et al. 2012, 2015; Zhu et al. 2015; Witt et al. 2016; Chen et al. 2017; Wang et al. 2020; Börner et al. 2022). Between approximately 1500 and 1750 ce, corresponding to the Little Ice Age (LIA), large fluctuations in geochemical variables indicate alternating humid and arid periods (Mügler et al. 2010; Wrozyna et al. 2010). During this time, the climate transitioned from wet‐cold to dry‐cold conditions, leading to increased lake salinity and influencing ostracod populations (Xie et al. 2009). Notably, peaks in ostracod populations with black shells coincided with periods of heightened sedimentary water dynamics, suggesting a response to environmental changes (Xie et al. 2009). In recent decades, modern hydrological data indicate a rising lake level in Nam Co (Mügler et al. 2010; Lazhu et al. 2016; Ma et al. 2016; Tong et al. 2020). This trend is associated with climate warming and increased monsoonal activity and glacier melt, leading to enhanced freshwater input into the lake (Wu and Zhu 2008). These hydrological changes have likely impacted ostracod assemblages, reflecting their sensitivity to hydrological variations, e.g., salinity, water level, and temperature. Most likely, these changes have also influenced intra‐species diversity. However, the genetic response to these ecosystem changes is harder to study and to predict, and little is known about the environmental effects on genetic diversity and adaptability of species to those environmental changes in Nam Co.

Genetic studies on ostracods from the TP are very limited. However, a notable study employed a metabarcoding approach to identify ostracods in surface sediment samples from Nam Co, demonstrating the efficacy of high‐throughput sequencing in assessing ostracod diversity in this region (Echeverría‐Galindo et al. 2021). Further research can help clarify species boundaries in groups where morphological identification is challenging due to phenotypic plasticity, cryptic species, and high endemism. Morphological traits related to salinity vary across taxa. While some ostracod species have been suggested as indicators for salinity changes based on their morphology (Berndt et al. 2019), in others, such as *Limnocythere inopinata* , no direct effect of salinity on valve ornamentation could be identified (Yin et al. 1999). The morphological appearance of *L. inopinata* was instead mainly affected by the genotype (Yin et al. 1999). This is especially relevant for ostracods on the TP, where taxonomic diversity remains incompletely assessed (Mischke 2012; Echeverría‐Galindo et al. 2021; Peng et al. 2021). These factors contribute to misidentifications and inconsistencies in taxonomic classification (Fürstenberg et al. 2015; Echeverría‐Galindo et al. 2021). Genetic studies, such as DNA barcoding and whole‐genome sequencing, allow for more accurate species identification and help resolve taxonomic uncertainties in ostracod assemblages (Kilikowska et al. 2024). Additionally, analyzing genetic diversity within and among ostracod populations can provide valuable insights into their yet still partly unresolved evolutionary history (Martens 1994; Jeffery et al. 2017), dispersal mechanisms (Danielopol et al. 1994; Chaplin and Ayre 1997), and responses to environmental changes (Rodriguez‐Lazaro and Ruiz‐Muñoz 2012). The Tibetan Plateau has undergone dramatic climatic changes over the past millennia, influencing the hydrology and ecology of Nam Co. Genetic studies could reveal how past climate shifts have shaped ostracod populations, identifying potential bottlenecks, local adaptations, or genetic divergence among isolated populations (Martens et al. 2008).

Moreover, genetic diversity can serve as an indicator of ecosystem health and resilience (Sjöqvist and Kremp 2016). High genetic variability within ostracod populations may suggest a greater ability to adapt to changing environmental conditions, while low diversity could indicate vulnerability to ecological stressors such as salinity fluctuations, temperature shifts, and habitat alterations due to climate change. By assessing genetic diversity over time, we can better predict how ostracod species—and the aquatic ecosystems they inhabit—may respond to future climate change scenarios (Rossetti et al. 2020).

The aims of this study were to use ostracod metabarcoding data and environmental variables of a previously studied sediment core (Anslan et al. 2022) from Lake Nam Co, Tibetan Plateau to: (1) reconstruct genetic species assemblage shifts of these important bioindicators during the last two millennia, (2) determine how species diversity and community structure have changed in response to climatic and hydrological fluctuations, and (3) investigate how major climate periods (Medieval Climate Anomaly, Little Ice Age, modern warming) influenced the genetic diversity.

## 2 Materials and Methods

### 2.1 Study Area

**FIGURE 1:**
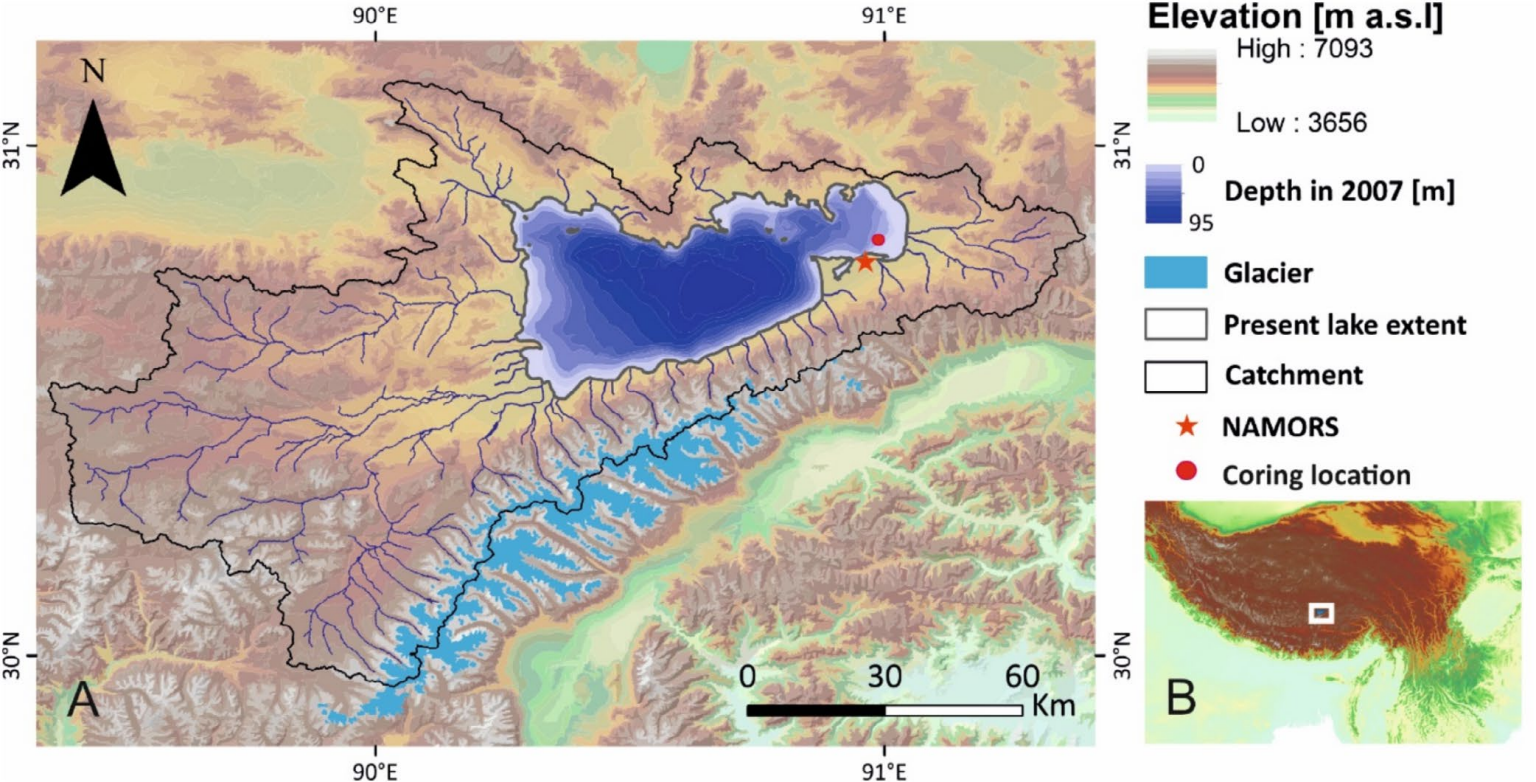
Coring location of NC19‐C8 within Nam Co (red dot) and surface elevation of the catchment including the lake extent based on Sentinel‐2 imagery of 2018‐01‐30 (Esri; Garmin; Copernicus Sentinel data [2018] processed by ESA; Jarvis et al. 2008). Bathymetric depth in 2005 based on Wang et al. (2009), outline of the catchment (following Keil et al. 2010), and the glaciers of the Nyainqêntanglha Range (GLIMS and NSDIC 2005, updated 2018) are represented following Anslan et al. (2020), and the location of NAMORS (red star). Map modified from Echeverría‐Galindo et al. (2021) (A). Location of Nam Co on the Tibetan Plateau (JAXA; Copernicus Sentinel data [2018] processed by ESA; Jarvis et al. 2008) (B).

Nam Co is the third largest lake on the central Tibetan Plateau with an area of 2018 km^2^. Nam Co is a dimictic lake, endorheic, and located at a high altitude (4723 m a.s.l.) (Keil et al. 2010) (Figure 1). Between 2006 and 2017, the mean annual temperature measured at the Nam Co Observation and Research Station (NAMORS) was −0.6°C, and the annual precipitation was between 291 and 568 mm (mean = 406 mm), with the majority occurring during the monsoon season from May to October (Anslan et al. 2020). The lake is characterized by a conductivity of 1296 μS cm^−1^ (2005–2008 average 1851 μS cm^−1^; Wang et al. 2009), a pH of 9.1 (both in situ measurements of the surface water column at the sampling site in September 2019), monsoon‐fed rivers in the eastern basin, and glacier‐fed streams from the large glacial areas in the southwestern catchment of the lake (Anslan et al. 2022).

### 2.2 Sampling

**FIGURE A1:**
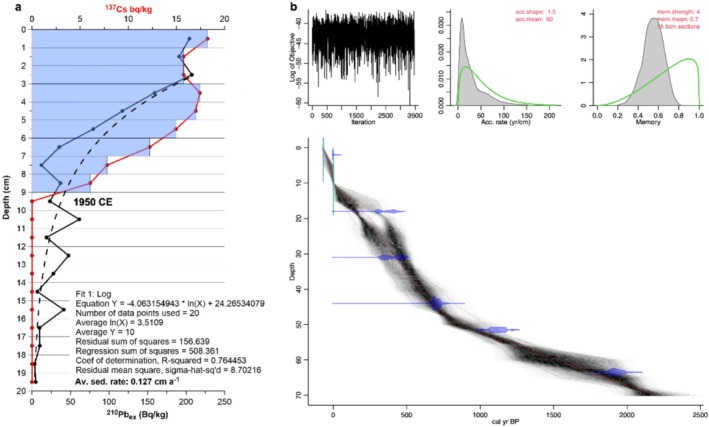
Age‐depth model of NC19‐C8 from Anslan et al. (2022). Built using 210Pb/137Cs dating from the top 20 cm of the core (a), and AMS C14 dating (b) based on the R algorithm in Bacon according to Blaauw and Christen (2011). The year 1950 ce likely corresponds to the core layer at 9.5 cm depth, 1963 ce at 7.75 cm depth and 1986 ce (Chernobyl nuclear outfall) at 3.5 cm depth.

The sampling of the 107‐cm‐long sediment core NC19‐C8, taken at a water depth of 27 m from the eastern part of Nam Co (30.812° N 90.992° E, altitude 4,723 m a.s.l.; Figure 1) in 2019, as well as the environmental conditions of Nam Co, have been described in Anslan et al. (2022) and the geochemical analysis (Anslan et al. 2022, Supplementary Table 1 therein) and the age‐depth model is also included therein (Appendix A, Figure A1).

### 2.3 Climate Periods

Climate periods referenced in the text and figures follow the definitions given in Appendix A—Table A1 (Start/End ages in Common Era (CE)). All time axes are plotted in CE, in stratigraphy with older ages at the bottom, in other plots with older ages on the left. Samples were assigned to climate periods using their median calibrated age.

### 2.4 Molecular Analysis

All molecular analyses were performed following the guidelines for sedaDNA laboratory work. Sample preparation, DNA extraction, and metabarcoding PCR were performed under a dedicated clean bench, which was sterilized with 5% bleach and UV light before and after work.

#### 2.4.1 DNA Extraction

Briefly, 95 samples for sedaDNA extractions were taken every centimeter along the core NC19‐C8 with sterilized spatulas and consisted of ~0.5 g of sediments (wet weight). Sterilization after each sample processing was done using 5% bleach, followed by an ethanol washing step and burning residual ethanol using a spirit burner. DNA was extracted using the DNeasy PowerSoil Kit (Qiagen, Germany). For details, refer to Anslan et al. (2022). Samples were extracted in two replicates and pooled. The DNA concentration was measured using the Qubit dsDNA High‐Sensitivity Assay Kit (Invitrogen). Samples were stored at −80°C until further processing.

#### 2.4.2 Amplification and Sequencing

We used the 18SV4osF and 18SV4osRs primers that amplify a nested ca. 118 bp fragment (Echeverría‐Galindo et al. 2021) of the nuclear gene for small subunit rRNA (18S). Primers were indexed with 8‐bp unique identifiers and 0‐ to 4‐bp heterogeneity spacers. Combinatorial indexing was used, where several unused combinations served as tag‐switching controls (Taberlet et al. 2018).

The total PCR mix volume per sample was 50 μL and consisted of 10 μL Hot Start FirePol Master Mix (Solis BioDyne, Estonia), 1 μL of forward and reverse primer (10 μM), and a template DNA concentration of 4 ng/mL. The rest of the volume was filled with nuclease‐free water. PCR cycling conditions were as follows: 95°C for 15 min; 50 cycles of 98°C for 20 s, 54°C for 45 s, and 72°C for 1 min; with a final extension at 72°C for 5 min. Two uniquely indexed PCR replicates per sample were amplified and checked using gel electrophoresis with 5 μL PCR product on a 1% agarose gel. The MinElute PCR Purification Kit (Qiagen, Germany) was used for the purification of all PCR products, following the manufacturer's protocol. Samples were pooled equimolarly for sequencing on an Illumina NextSeq 550 system, using the high‐output kit to generate 2 × 150 bp paired‐end reads. Raw sequencing data have been deposited in the Sequence Read Archive (SRA) under BioProject PRJNA781478 (accession numbers: SRR33514267‐SRR33514365).

### 2.5 Sequencing Data Processing and Amplicon Sequence Variant Table Curation

The raw sequence data processing followed Anslan et al. (2022). Briefly, the bioinformatics were performed in PipeCraft2 (v0.1.0; Anslan et al. 2017) platform following DADA2 (Callahan et al. 2016) pipeline for generating amplicon sequence variants (ASVs). EMBL v143 (Kanz et al. 2005) and barcoded ostracod species from Nam Co by Echeverría‐Galindo et al. (2021) served as reference databases for the blastn search in the taxonomy assignment step. Potential tag‐switching errors were manually corrected using the unused index combination controls based on the relative abundances of the reads in these control samples.

Tag‐switch filtering removed 0.002% (1379) sequences from the ostracod ASV table. No sequences were present in the PCR negative controls, and only one ASV (assigned to bacteria) was detected in the 4 DNA blank extraction samples, indicating no obvious (cross‐) contamination. The majority of the sequences (97.7%) in the filtered ostracod ASV table were assigned to Ostracoda. The remaining 2.3% of reads were assigned to bacteria, plants, other arthropods, or were unassigned. Followingly, the ASV table was filtered to include ASVs that were assigned as ostracods (similarity and query coverage of ≥ 95% against a reference sequence) and had expected sequence length (118 ± 3 bp). The filtered Ostracoda ASV table was subjected to a post‐curation process using LULU (Frøslev et al. 2017) by setting the minimum match threshold to 99%.

The taxonomic assignment was based on the available information included in the EMBL v143 reference database. In the case of *Tonnacypris gyirongensis*, which has been synonymised under *Tonnacypris stewarti* (Bonilla‐Flores et al. 2024), we used *Tonnacypris gyirongensis* throughout the manuscript to stay consistent with the genetic reference database.

### 2.6 Statistics

**FIGURE B1:**
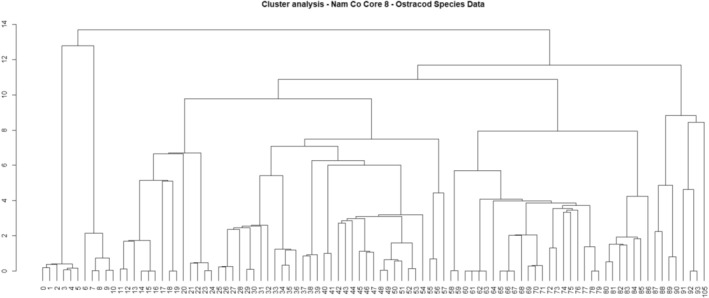
Constrained cluster analysis of ostracod species data from Nam Co core NC19‐C8. The cluster analysis was constrained based on the stratigraphic order of the samples. Numbers on the x‐axis indicate the sample depth in cm.

**FIGURE B2:**
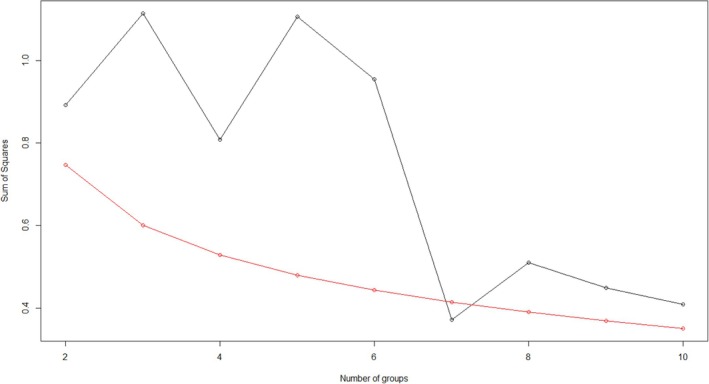
Broken‐stick plot of the ostracod data.

Statistical analyses were carried out using the *vegan* package version 2.5–7 (Oksanen et al. 2015) in R version 4.1.0 (R Core Team 2021). Environmental variables were log‐transformed, while species composition data were Hellinger‐transformed using *vegan*. Temperature and precipitation data were standardized using the scale() function in R. To calculate the biostratigraphic units as accurately as possible, a constrained hierarchical clustering of a distance matrix with the CONISS algorithm using chclust was performed using the *rioja* package version 0.9–26 in R (Juggins 2015). The dispersion of the hierarchical classification was compared to a broken‐stick model (Bennett 1996), in order to determine the number of biostratigraphic zones with the function bstick. Figures of cluster analysis and broken‐stick modeling can be found in Figures B1 and B2. All samples were included in the cluster analysis and broken‐stick model; however, due to sediment disturbance, the age‐depth model was only reliable to a depth of 71 cm, although the sediments from ~50–71 cm contain some uncertainties. Therefore, the stratigraphic plots only represent the reliably dated core sequence. The stratigraphic plots were implemented in C2 version 1.7.7 (Juggins 2007). CorelDRAW Graphics Suite 2021 (Version 23.1.0.389) was used to polish figures. For further analysis of the ostracod ASVs, we chose a read count cut‐off of 100, because most sequence reads in this dataset belong to only two ASVs. However, the known ostracod diversity in Nam Co is limited to comparatively few species, and we wanted to include the highest possible genetic diversity; thus, we selected the rather low read count cut‐off. After filtering for a minimum of 100 reads, we only kept 22 ASVs identified to at least genus level.

#### 2.6.1 Diversity Metrics

The following α diversity and β temporal diversity metrics were calculated for data analysis using the *codyn* package version 2.0.5 (Hallett et al. 2020) in R. For α diversity: Richness—measures the number of species or genetic variants (ASVs) in a community; Simpson Index—reflects the probability that two ASVs randomly selected from a sample belong to the same species; Simpson Evenness—captures how evenly ASVs are distributed across species and is derived from the Simpson Index. For β temporal diversity: Turnover—reflects the rate of species or ASV replacement over time (calculated proportion of species that differ between consecutive time points; summed appearances and disappearances relative to total species richness), providing insights into community stability or shifts. We combined these indices to provide a more comprehensive understanding of the community diversity (Details in Appendix C—Tables C1 and C2).

#### 2.6.2 Phylogenetic Diversity

Phylogenetic diversity (PD) was calculated using the *picante* package version 1.8.2 (Kembel et al. 2010) in R. Before running the analysis, sequences were aligned with MAFFT v. 7.490 (Katoh and Standley 2013) through the L‐INS‐i approach. Consequently, phylogenetic trees were inferred through the software IQ‐TREE v2.3.6 (Minh et al. 2020), including the selection of the better‐fitting substitution model (Kalyaanamoorthy et al. 2017) and the Ultrafast Bootstrap functionality UFBoot (Hoang et al. 2018). As an outgroup, the 18S ribosomal RNA gene of *Daphnia pulex* (NCBI accession number: AF014011) was added to the alignments. Finally, to normalize the data before calculating the phylogenetic diversity, a sample by species matrix was created, with the abundance of each ASV across the sample.

## 3 Results

### 3.1 Ostracod Assemblage Composition

The genetic raw data counted 734,314 reads, consisting of 437 sequence types (ASVs). Overall, in all four DNA extraction blanks, 188 sequence reads were retrieved. Despite the equimolar pooling of PCR products for multiplex sequencing (i.e., samples are tagged and pooled for DNA quantity in each sample, allowing the sequencing of different samples in one NGS run), the total number of sequence reads after filtering for ostracod sequences varied substantially among samples, i.e., between 30,664 and 132, with a median of 5799. Among total counts, *Leucocytherella sinensis* (515,407) and *Limnocythere* sp. (185,520) were most often recorded. Similarly, the number of retrieved ASVs was highest in *Leucocytherella sinensis* and *Limnocythere* sp., with 11 and 4, respectively, followed by every two ASVs belonging to Cyprididae (5802 total reads) and *Candona candida* (1000 total reads). Regarding taxonomic resolution, in the ostracod genus *Limnocythere*, only *L. inopinata* could be identified to species level (274 total reads, one ASV). All other ASVs in this genus were labeled *Limnocythere* sp., *Ilyocypris angulata*, and *Tonnacypris gyirongensis* were each represented by one ASV and 5574 and 174 total reads, respectively.

### 3.2 Temporal Taxonomic Composition

**FIGURE 2:**
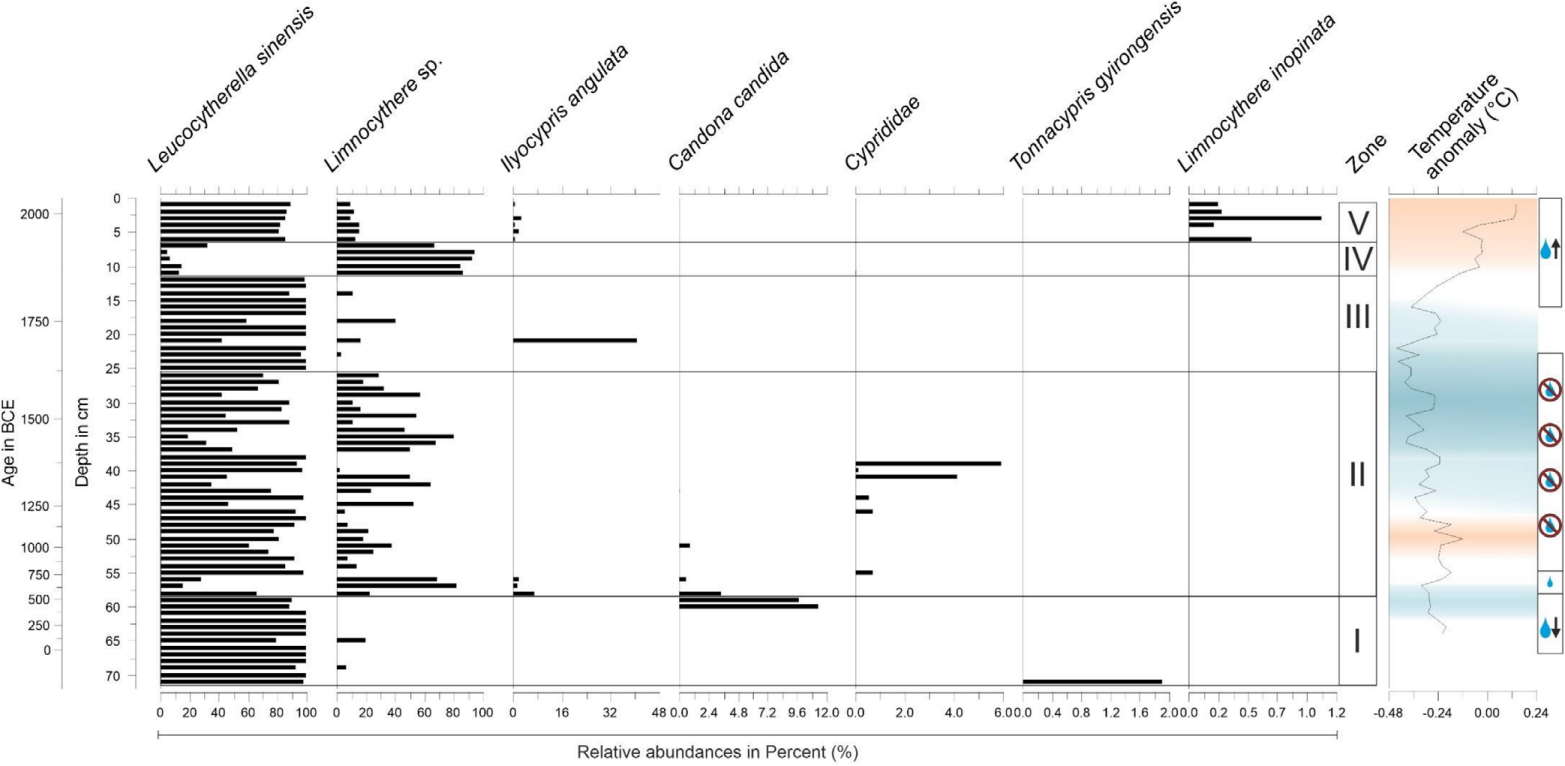
Stratigram of relative abundances of identified ostracod taxa through ASV assignment in the Nam Co sediment core. As described in Anslan et al. (2022), the temperature data between 1000 and 1980 ce included the reconstructed Northern Hemisphere (NH) mean surface temperature anomaly from Mann and Jones (2003). The global mean temperature change after 1980 was integrated with the NH mean surface temperature anomaly using linear interpolation to extend the time series for the whole time period of the sediment core (Collins et al. 2013). Temperature anomaly data per sediment sample correspond to the top of the layer age. The blue and orange shadings indicate the warm and cold periods, from bottom to top: Late Antique Cold Period, Medieval Climate Anomaly, Little Ice Age, Recent Global Warming (for details see Appendix A—Table A1). The graphical indicators for low lake levels (drop with downward arrow), moist conditions (drop), dry conditions (drop with red crossed‐circle), and moist with increasing precipitation and rising lake levels (drop with upward arrow) represent the lake level and precipitation changes reported from Nam Co in Kasper et al. (2012) and Wrozyna et al. (2012).

Five biostratigraphic units were identified along the core in the ostracod assemblage (OA) using cluster analysis (Figure 2).

#### 3.2.1 OA Zone I (70 cm–58 cm = ~230 BCE–560 ce)

Zone I is dominated by *Leucocytherella sinensis* with an average of ~93.71% per sample depth. The second most abundant group is ASVs belonging to *Limnocythere* sp., which appeared sporadically throughout Zone I at 68 cm (~6.7%) and at 64 cm (~20%). *Tonnacypris gyirongensis* occurs only at a depth of 70 cm with 1.91%. This is also the only record of this species throughout the entire core sequence. Towards the top of Zone I, *Candona candida* appeared with an abundance peak of 11% at 60 cm depth and declined towards the beginning of Zone II. Similarly, *Ilyocypris angulata* started appearing at this interface between Zone I and Zone II at 58 cm depth and with an abundance of ~7%.

#### 3.2.2 OA Zone II (58 cm–25 cm = ~560 ce–1615 ce)

The beginning of Zone II is defined by the highest species diversity within this biostratigraphic unit. From 57 cm to 55 cm occurs *Candona candida* (~0.6%), Cyprididae (~0.74%), *Ilyocypris angulata* (~1.5%–1.9%), as well as the two dominating species *L. sinensis* (average abundance of 36.81%) and *Limnocythere* sp. (average abundance of 58.26%). Zone II shows more abundance shifts than Zone I. *L. sinensis* is still the most abundant species; however, in depths with lower abundance of this species, *Limnocythere* sp. shows the highest abundances. Additionally, Cyprididae occur again at 45 cm (~0.73%), 43 cm (~0.6%), and from 40 to 38 cm (~4.2%, 0.14%, and 5.93%).

#### 3.2.3 OA Zone III (25 cm–11 cm = ~1615 ce–1926 ce)

Zone III is mainly characterized by the two dominant species *L. sinensis* (average of 91.28%) and *Limnocythere* sp. (average of 10.64%), with the sole exception of 20 cm depth, where *I. angulata* had an occurrence of ~40.71%.

#### 3.2.4 OA Zone IV (11 cm–6 cm = ~1926 ce–1971 ce)

The transition from Zone III to Zone IV is dominated by a high abundance of *Limnocythere* sp. and a low abundance of *L. sinensis* . Throughout Zone IV *Limnocythere* sp. occurs on average with 89.69%, while the relative abundance of *L. sinensis* increases from 10.29% in the lower depths of Zone IV to 32.66% at 6 cm depth.

#### 3.2.5 OA Zone V (6 cm–0 cm = ~1971 ce to the present)

In Zone V, *L. sinensis* continues as the dominant species (on average 85.37%), whereas *Limnocythere* sp. decreases drastically from 67.34% at 6 cm depth to 13.21% at 5 cm depth. This shift between *L. sinensis* and *Limnocythere* sp. marks the transition between Zones IV and V. Throughout Zone V *Limnocythere* sp. makes up 12.94% of the ostracod assemblage. This Zone is the only biostratigraphic unit with an occurrence of *L. inopinata* , which has a relative abundance of 0.39% throughout all sampled layers. *I. angulata* appears again with a relative abundance of ~1.3% throughout Zone V.

### 3.3 Changes in Species Richness, Temporal Diversity and Community Change

**FIGURE 3:**
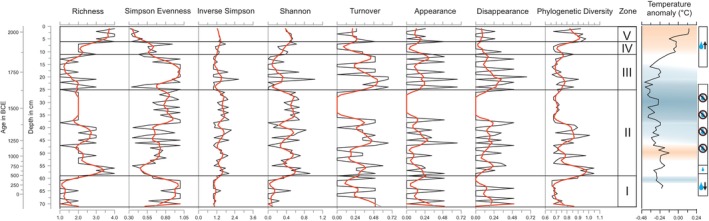
Temporal ostracod diversity changes derived from different diversity indices. Red line = mean trend. Details regarding the temperature anomaly plot, see the caption of Figure 2.

Five diversity indices were used to evaluate the temporal diversity changes in the ostracod community data. The calculated proportion of species that differ between time points (turnover) is also shown as appearances and disappearances (Figure 3, Appendix C—Tables C1 and C2).

#### 3.3.1 Temporal Diversity Changes in Ostracods

The ostracod species richness across the five biostratigraphic zones varied throughout the record (Figure 3). The mean species richness was lowest in Zone I (mean = 1.38) and increased at 628 ce (end of the Late Antique Cold Period (LACP)) with the beginning of Zone II (mean = 2.3). Richness decreased throughout this zone and plateaued from 1436 to 1615 ce (corresponding with the Little Ice Age on the Tibetan Plateau). In Zone III, species richness varied a lot with a mean of 1.5, and increased with less variation in Zone IV (mean = 2.2). However, Zone V showed the highest ostracod species richness (mean = 3.6). The Simpson Evenness gives a diversity value between 0 and 1; the larger the value, the lower the diversity. This index shows, similar to the species richness, that Zone I (mean = 0.85) is low in ostracod diversity, while the diversity slightly increases in Zone II (mean = 0.71). Zone III (mean = 0.85) has an overall equally low ostracod diversity, whereas the diversity increases through Zone IV (mean = 0.63) all the way into Zone V (mean = 0.37). The same pattern can be seen with the Inverse Simpson index (Zone I: mean = 1.09, Zone II: mean = 1.53, Zone III: mean = 1.21, Zone IV: mean = 1.34, and Zone V: mean = 1.34) and the Shannon diversity index (Zone I: mean = 0.12, Zone II: mean = 0.48, Zone III: mean = 0.16, Zone IV: mean = 0.39, and Zone V: mean = 0.47). Species turnover varied in Zone I (mean = 0.27), alternating between phases of no turnover and appearances and disappearances. A lot of species turnovers occurred in Zone II (mean = 0.21); important phases are the Medieval Climate Anomaly (MCA) and the LIA. Higher species turnover occurred in Zone III (mean = 0.42), while it decreased in Zone IV (mean = 0.13) and increased again in Zone V (mean = 0.25).

Generally, the MCA is characterized by multiple changes in ostracod species richness and various turnover events. During the LIA and its short recurrence in the early 1800s, species turnover was near 0 and 0.25, respectively, with a 0.5 species disappearance during the beginning of the short recurrence. Species richness during these two events is low, with 2 and 1, respectively.

#### 3.3.2 Phylogenetic Diversity Changes

Phylogenetic diversity varied slightly over the last ~2250 years, with larger, but more stable changes throughout the period from 230 years BCE to ~1000 years CE, and notably more and rapid changes throughout the last millennium (Figure 3). The mean PD across all samples was 0.766 ± 0.11, with the highest PD recorded at 628 years CE (1.05) and the lowest at 424 years CE (0.66) (Figure 3), both falling within the LACP.

**FIGURE 4:**
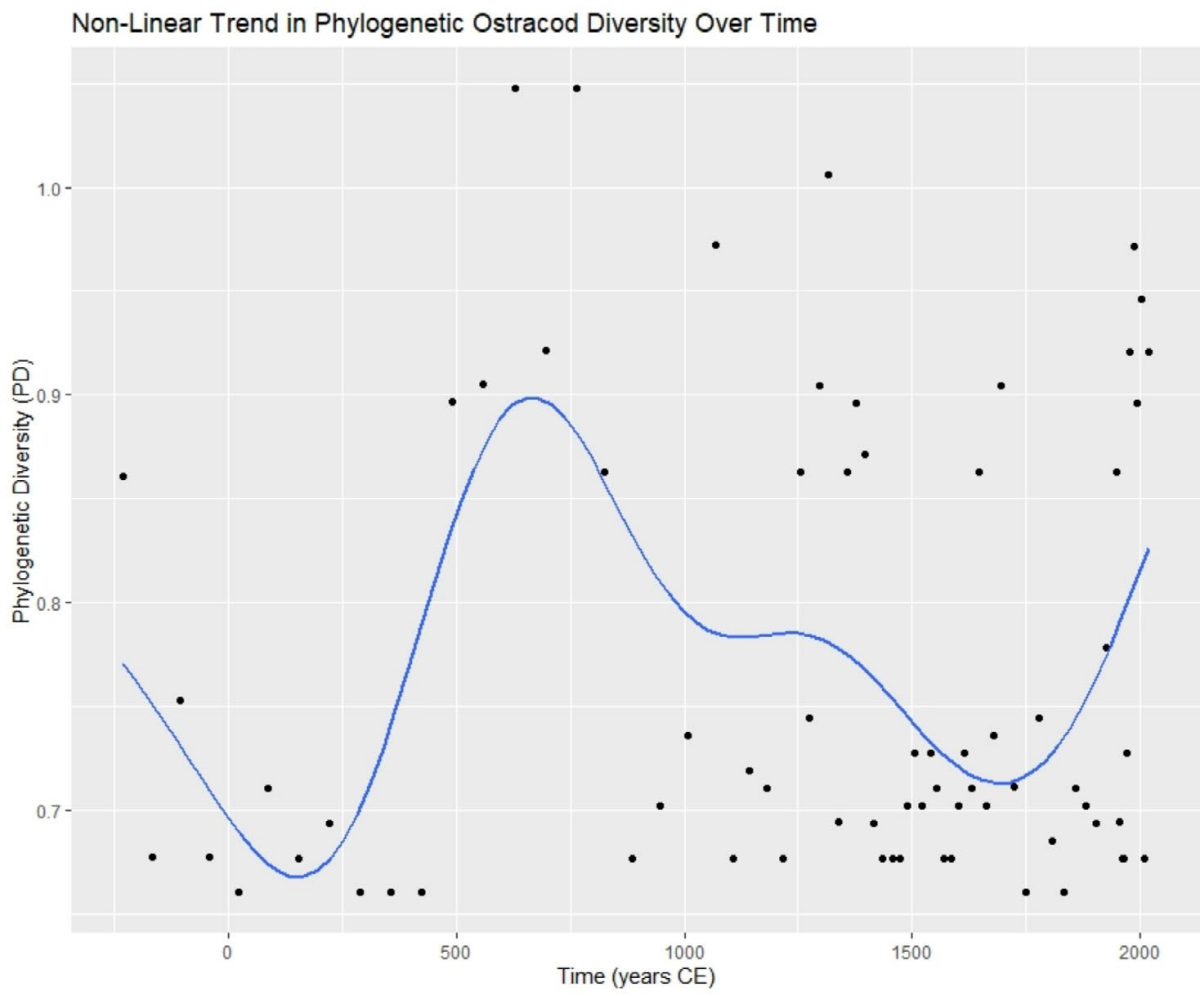
Generalized additive model of the non‐linear relationship between phylogenetic diversity (PD) of the ostracod ASV data recorded across the sediment core spanning over two millennia. Blue line = mean trend.

**FIGURE D1:**
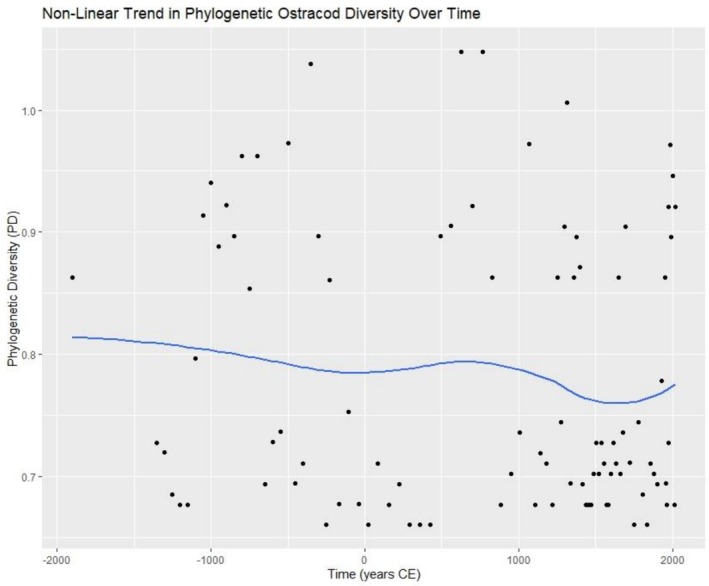
LOESS model of the non‐linear trend in phylogenetic ostracod diversity over time. All samples included.

The PD of ostracod data displayed a non‐linear trend over the past ~2250 years, with the highest PD values recorded during the periods from ~490–830 years CE, ~1250–1400 years CE, and with the exception of 2010 from ~1975–2019 years CE. Shorter periods of high phylogenetic diversity were recorded in 1070 ce, 1647 ce, 1694 ce, and 1949 ce (Figure 3). A generalized additive model indicated a significant, non‐linear relationship between PD and time (*F* = 2.487, *p* = 0.018) (Figure 4). Results of the undated core section can be found in the Figure D1.

**FIGURE 5:**
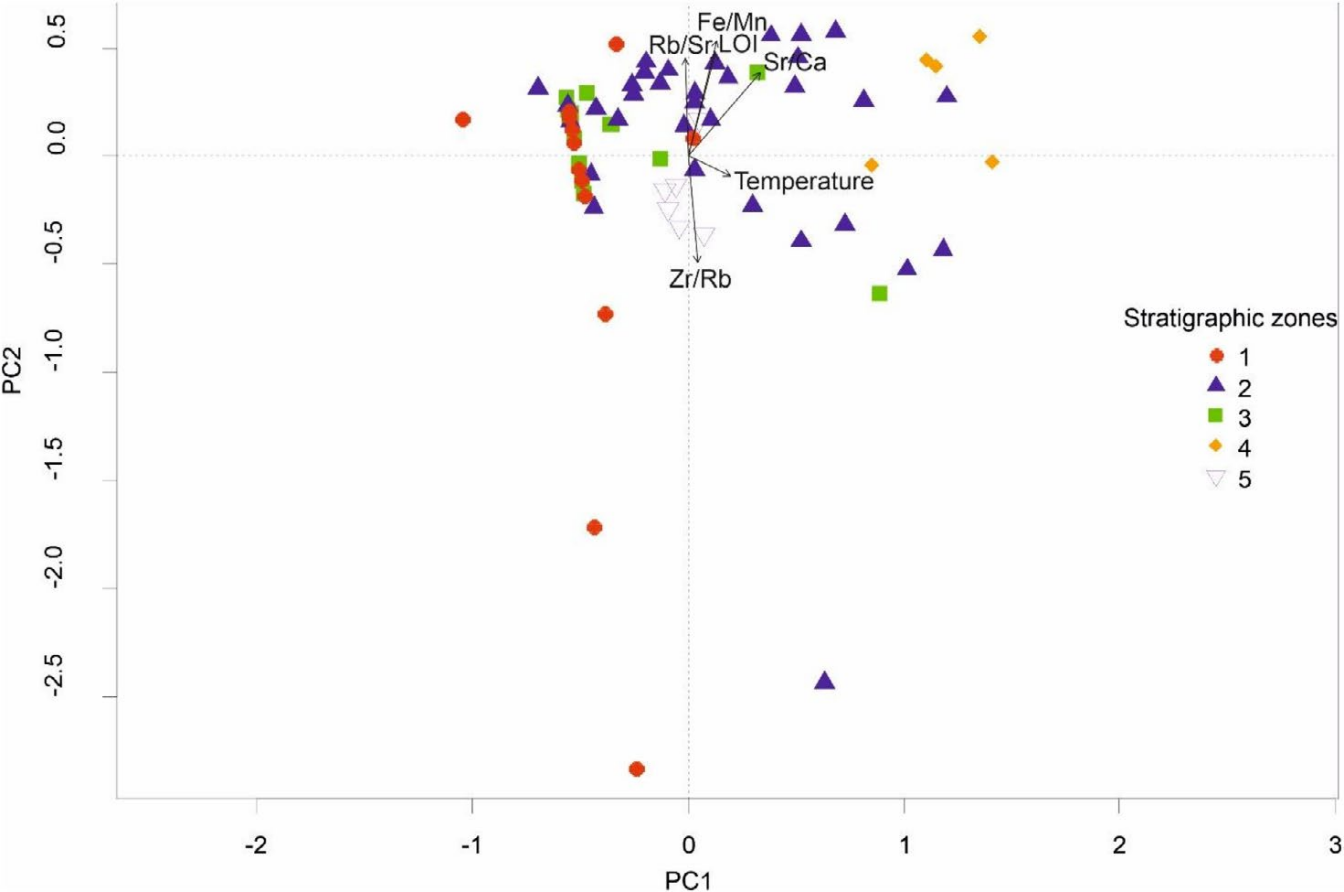
Principal Component Analysis of the ostracod ASV data from Nam Co core NC19‐C8 with environmental gradients plotted using envfit. Samples are color and shape‐coded based on the stratigraphic zones corresponding to Figure 2.

Transformation‐based RDA (redundancy analysis) ordination was used to test which of the environmental variables explains the compositional changes of the ostracod assemblages. The proportion of the total variance, which is explained by all environmental factors, is 1.78% (*F* = 1.091, *p* > 0.367), which is negligible. Thus, a forward selection to identify the environmental variables that explain a significant portion of the variance was not possible. The first constrained axis of the then run Principal Component Analysis (PCA) explained 24.91% and the second constrained axis explained 18.52% (Figure 5). Even though no significant environmental variables could be identified as explanatory for the ostracod assemblage, the known factors influencing ostracods were plotted as well (Figure 5). These indicate a temperature gradient for PC1 and a “moisture” gradient based on weathering, runoff, and salinity for PC2.

## 4 Discussion

Our findings show that ostracod diversity varied throughout the last two millennia in Nam Co, as indicated by stratigraphy, temporal diversity indices, and phylogenetic diversity. Ostracod species richness peaked from 628 ce to 765 ce (end of LACP) and reached another peak since 1976 ce (Figure 3). Interestingly, Mischke and Zhang (2010) reported that the LACP had probably no severe and widespread impacts in the region, as it was not recorded in a number of sites. The period since 1976 is characterized by several turnovers with both appearances (ASVs of *Ilyocypris angulata*, *Limnocythere inopinata* ) and disappearances (*Leucocytherella sinensis* ASVs) in the species composition.

### 4.1 Lake Level Shifts and Species Diversity

**FIGURE 6:**
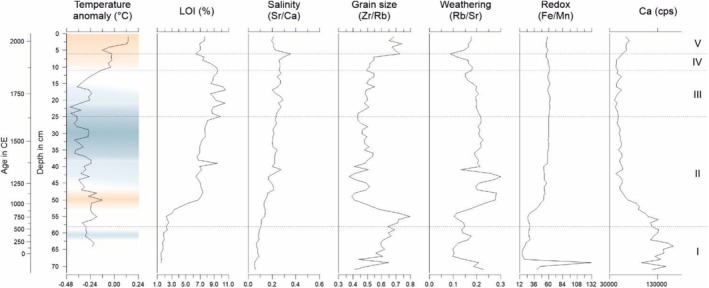
Environmental variables from Anslan et al. (2022) supplementary table 1 and the temperature anomaly (data described in the caption of Figure 2) for Nam Co.

Previous studies of Nam Co have identified significant lake level shifts throughout the past two millennia and beyond (Daut et al. 2010; Frenzel et al. 2010; Mügler et al. 2010; Wrozyna et al. 2010; Kasper et al. 2012; Doberschütz et al. 2014). The identified shifts of moist and dry phases, associated with lake level rise or lowering from Kasper et al. (2012) can also be linked to our findings regarding genetic diversity changes in the ostracod communities. The presence of *Tonnacypris gyirongensis* at the bottom of our sediment archive (~30 BCE) indicates warm‐nutrient rich surface waters, a shallow littoral zone, and estuary‐like waters (Akita et al. 2016), which indicates lower lake levels compared to the most recent samples at the top of the core. This is also indicated by the PCA that shows the potential influence of run‐off (grain size—Zr/Rb) on biostratigraphic Zone I (Figure 6). During the period of moister conditions or possibly less vegetation cover increasing runoff and minerogenic input (Kasper et al. 2012) between ~600 ce and ~830 ce (corresponding to 1480 cal. BP and 1200 cal. BP in Kasper et al. 2012), we could observe the periods with increased ostracod richness. During this period, *Candona candida* and *Ilyocypris angulata* are present in the retrieved assemblage, both indicating shallow and freshwater, with high organic matter content (Akita et al. 2016). The period of the Medieval Climate Anomaly, as well as the Little Ice Age, are dominated by *Limnocythere* spp. and *Leucocytherella sinensis*. Their shift in dominance is primarily influenced by changes in lake level and salinity. Dominance of *L. sinensis* is associated with higher lake levels and lower salinities, whereas *Limnocythere* spp. may indicate higher salinities and lower lake levels (Alivernini et al. 2018). The direct (salinity (Sr/Ca)) and indirect environmental effects (weathering (Rb/Sr) and redox conditions (Fe/Mn)) on the ostracod assemblage during these time periods are reflected in Zones II and III (Figures 5 and 6).

**FIGURE 7:**
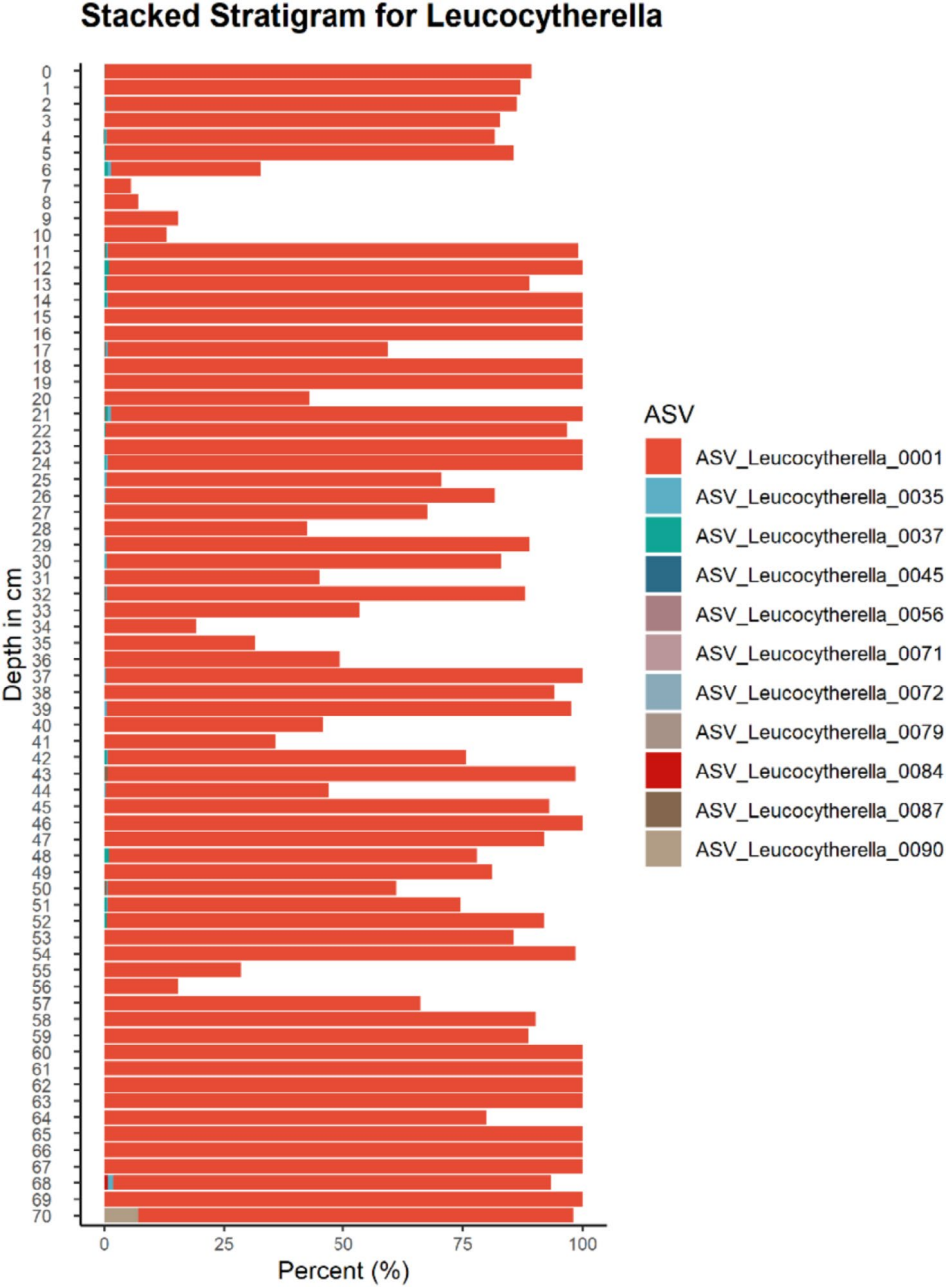
Stratigram showing sample‐specific ASV proportions of Leucocytherella sinensis.

During the beginning and end of the MCA, our data also shows some intra‐species variation in ASVs, especially within *L. sinensis* (Figure 7, Appendix E). Additionally, the analyzed species diversity shows significant variation, especially since the 1970s (e.g., increasing ostracod species richness but decreasing alpha diversity and decreasing phylogenetic diversity (Figure 4)). This is most likely influenced by the lake level rise due to increasing glacier melt and changes in the monsoon‐fed rivers and streams, and thus affecting the salinity (lower Sr/Ca) and alkalinity of the lake. This water level rise changes the salinity, and air temperature changes affect the period of ice cover, which in turn both influence the ostracod assemblages that are sensitive to light, water temperature, and salinity. The appearance of *L. inopinata* and *I. angulata* in Zone V indicates an increase in organic matter content and brackish to fresh water (Figure 5). This coincides with the drastically increasing lake level since the 1990s (Zhang et al. 2021).

As has previously been reported based on morphological data, the ostracod assemblage in Nam Co is characteristically dominated by *Leucocytherella sinensis* Huang et al. (1982), and *Leucocythere dorsotuberosa* Huang et al. (1982) (Wrozyna, Frenzel, Xie, et al. 2009; Mischke 2012). These are clustered in our genetic data as *L. sinensis* due to a lack of *L. dorsotuberosa* sequences in the reference database. *L. sinensis* is the most abundant and endemic ostracod found on the TP and is reported to account for up to > 98% of ostracod community assemblages (Fürstenberg et al. 2015), as seen also in the here presented data. The species displays various morphological forms (Fürstenberg et al. 2015); however, even though we retrieved multiple ASVs that potentially could reflect morphological diversity, one ASV is dominating the data. Yet, from 948 to 1143 ce (warm period; Zhu et al. 2008; Li et al. 2011), 1316 to 1554 ce (end of MCA; Ge et al. 2013), 1600 to 1678 (LIA; Huang et al. 2023), 1857 to 1926 ce (transition from the last minor cold phase to warmer climate; Hochreuther et al. 2015), and 1971 to 2019 (recent global warming; Huang et al. 2023), small proportions of diverse *L. sinensis* ASVs are present (Figure 6).

Even though this cold‐water adapted ostracod assemblage in cold glacial lakes, such as Nam Co, is low in species diversity (7 taxa), genetic diversity changes could be observed. However, the stratigraphic analysis of retrieved ostracod ASVs indicates limited nuclear diversity, which is likely due to parthenogenetic reproduction and its limited mutation rates in most of the retrieved taxa. The complex evolutionary history of ostracods and the frequently and independently occurring asexual reproduction (Schön and Martens 2016) makes further research on this topic extremely interesting. The periods of highest Phylogenetic Diversity can be associated with the period of moister conditions (~490–830 years CE—high PD, ~600 ce, and ~830 ce—moister conditions, Kasper et al. 2012), the Medieval Climate Anomaly (~1250–1400 years CE—high PD, ~1150 ce—1300 ce—warmer conditions, Ge et al. 2013), and the recent global warming (since the 1970s, Huang et al. 2023) in the catchment of Nam Co and the general central southern Tibetan Plateau.

### 4.2 Medieval Climate Anomaly and Little Ice Age

The ostracod data show high turnover rates towards the onset and end of the MCA, but a rather stable ostracod community composition during the MCA. In the remnant of the MCA Cyprididae ASVs appear and peak around 1400 ce (Figure 2). Reports of timing and extent of the Little Ice Age (LIA) vary across the TP (Xu and Yi 2014). The eastern Nyainqêntanglha Range, located at the southern shores of Nam Co, is a key region impacted by climate change, as it is one of the most heavily glaciated areas on the TP (Hochreuther et al. 2015). Climatic changes during the LIA have been studied on tree ring data from glaciers of the Nyainqêntanglha mountains (Hochreuther et al. 2015) and are thus used as reference for observed assemblage changes within Nam Co. The richness of the ostracod assemblage shown in the Nam Co sediment core appears stable during the LIA. However, drastic changes within the community can be observed between ASVs matched to *Limnocythere* sp. and *Leucocytherella sinensis*, which is reflected in the Shannon and Simpson diversity metrics. These dominance changes might be indicators for salinity changes in Nam Co, as *L. sinensis* is known to dominate Ca‐depleted brackish waters (Akita et al. 2016), whereas *Limnocythere* sp. might indicate changes in salinity and alkalinity, as some *Limnocythere* spp. can be restricted to certain salinity ranges (Jiang et al. 2022). More species‐specific reference genes in the database would help elucidate these ecological changes in more detail if we could match the *Limnocythere* sp. ASVs to species level. This period is characterized by cold and wet climate between 1700 and 1500 years BP based on pollen data (Zhu et al. 2008). The retreat of the first glaciers (~1740 ce) at the end of the last major cold phase (Hochreuther et al. 2015) potentially coincides with the appearance of *Ilyocypris angulata*. ASVs clustered as *L. sinensis* are the only ostracods retrieved during the last minor cold phase of the LIA on the TP (1790–1860 ce, Hochreuther et al. 2015).

### 4.3 Recent Global Warming

From ~1950 to the early 1970s, a drastic decrease in the previously dominating *Leucocytherella sinensis* types and an increase in *Limnocythere* sp., which in turn became the dominating ASVs in the ostracod assemblage, can be observed in the genetic data. This coincides with the observed decreased run‐off temporarily stalling the lake expansion (Kang et al., forthcoming). Though in the morphological data from the same core, no *Limnocythere* sp. were retrieved in the corresponding layers (Echeverría‐Galindo pers. comm.). Therefore, the ASVs clustered as *Limnocythere* sp. might be misidentified in the reference database. However, since the early 1970s up to 2019, *Leucocytherella sinensis* increases again to an average proportion of ~85%. *Limnocythere* sp. drops down to an average proportion of ~13%, and ASVs identified as *Ilyocypris angulata* and *Limnocythere inopinata* appear in the assemblage in small proportions. The appearance of *L. inopinata* coincides with the temperature increase due to climate change. The species turnover and following increase in species diversity in the ostracod community from the 1950s to 2019 is most likely influenced by lake freshening through increased rainfall due to a northward shift of the ITCZ, as well as milder temperatures, increased melt water, warmer lake water temperatures, and a decreased winter ice cover, compared to previously more saline and colder conditions (Kang et al., forthcoming). However, comprehensive data on the ecology of the retrieved ostracod species is lacking in order to give a more detailed interpretation of the observed diversity changes.

Generally, our data showed very little intra‐species variation, which might be due to the applied nuclear marker (Schön et al. 2014) that was selected due to its efficacy in retrieving the most consistent results compared to morphological data (Echeverría‐Galindo et al. 2021). This implies that future metabarcoding studies wanting to explore intra‐species diversity and detect cryptic species should be careful about their primer selection. Especially in a paleoenvironmental context, such as in the presented study, a multi‐primer approach might be advisable. As this study showed, aiming for the most comparable sedaDNA genetic assemblage data might miss intra‐species variation. Thus, a combination of the efficient 18S primers (Echeverría‐Galindo et al. 2021) and, e.g., the mitochondrial 16S region (Schön et al. 2014) could offer a holistic approach to species and intra‐species diversity in past ostracod populations.

## 5 Conclusions

**FIGURE E1:**
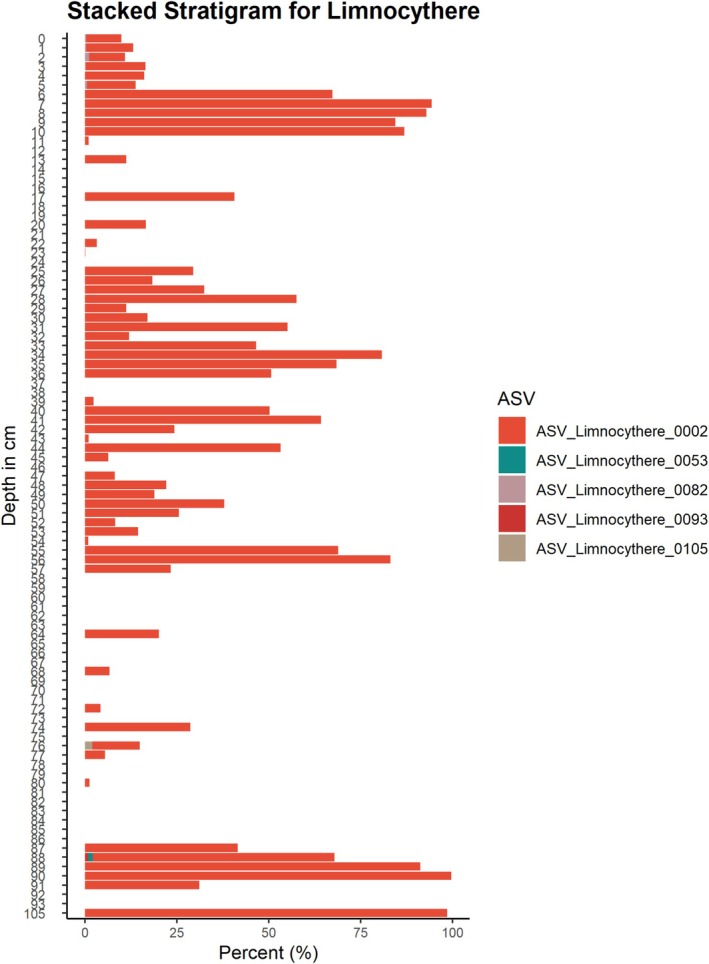
Stratigram showing stacked proportions of ASVs matched to Limnocythere.

**FIGURE E2:**
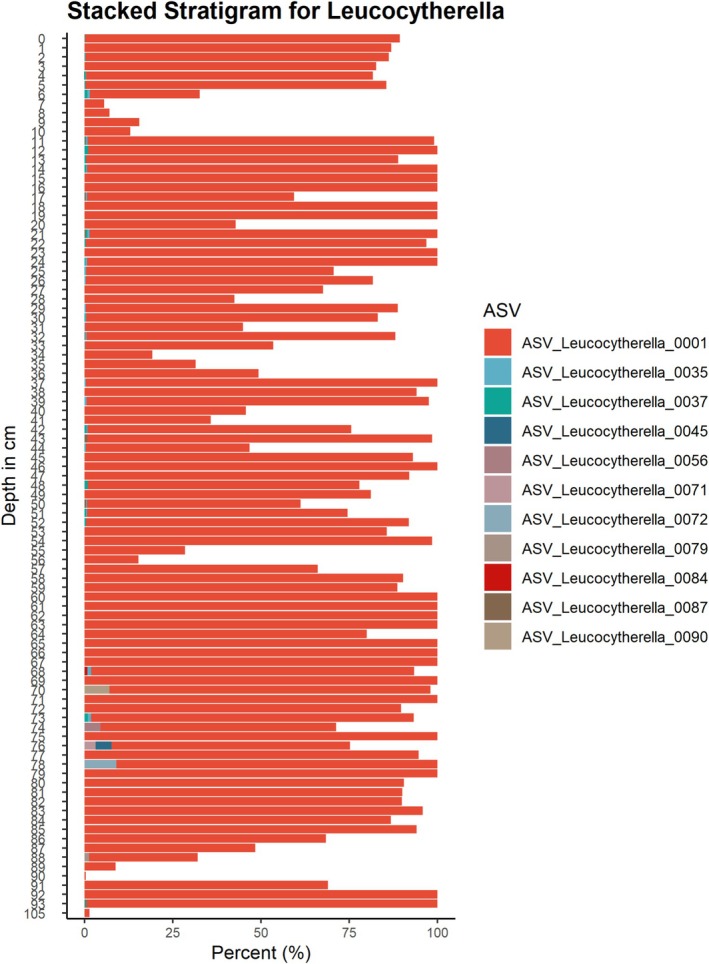
Stratigram showing stacked proportions of ASVs matched to Leucocytherella.

**FIGURE E3:**
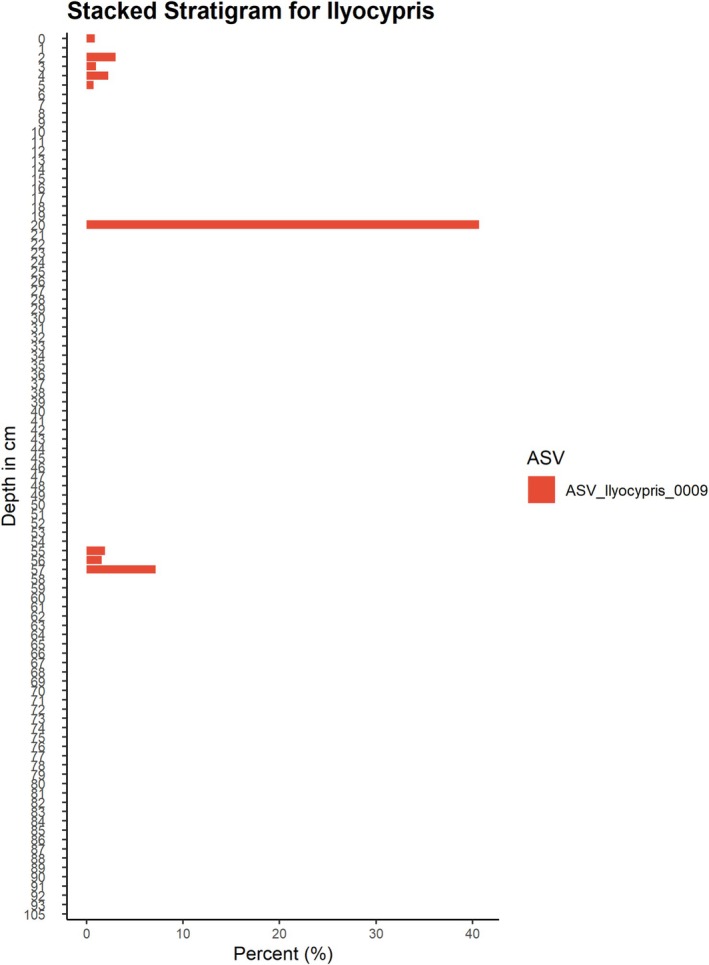
Stratigram showing stacked proportions of ASVs matched to Ilyocypris.

**FIGURE E4:**
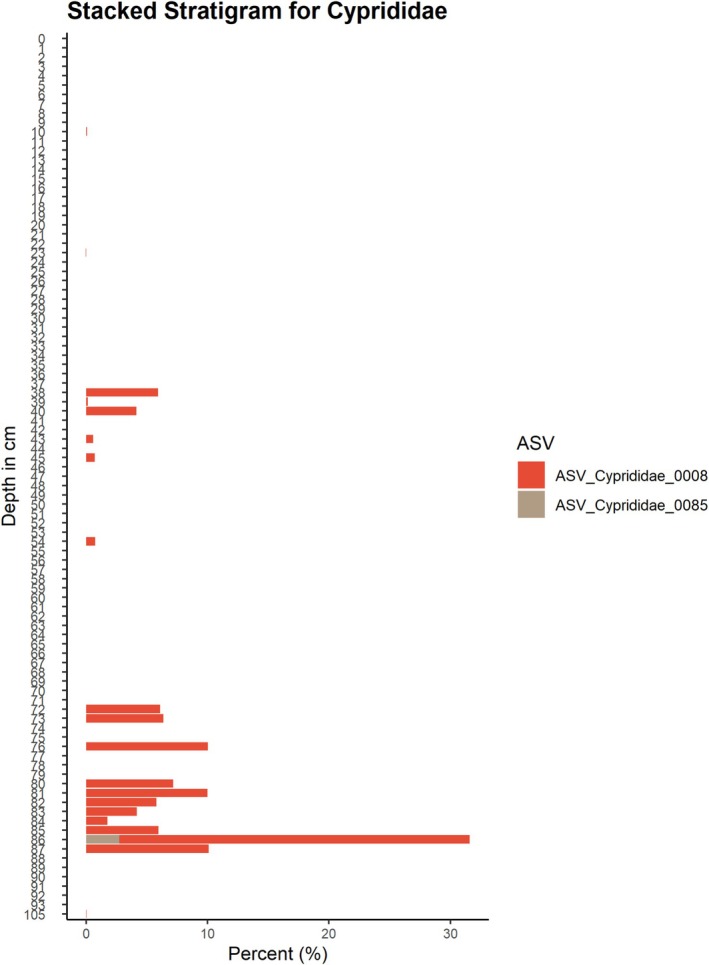
Stratigram showing stacked proportions of ASVs matched to Cyprididae.

**FIGURE E5:**
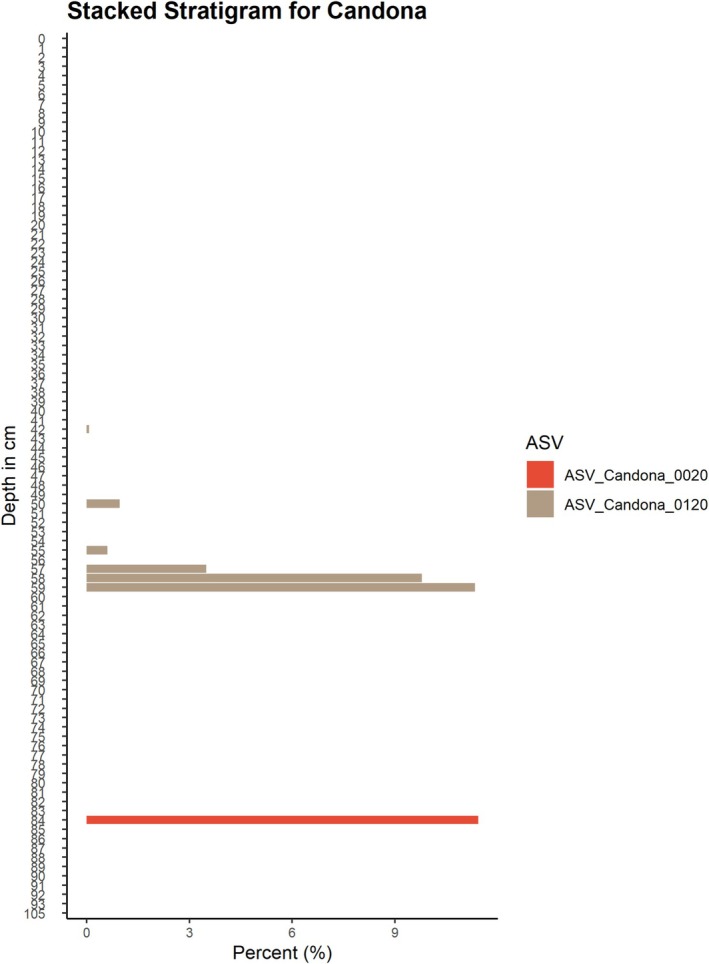
Stratigram showing stacked proportions of ASVs matched to Candona.

**FIGURE E6:**
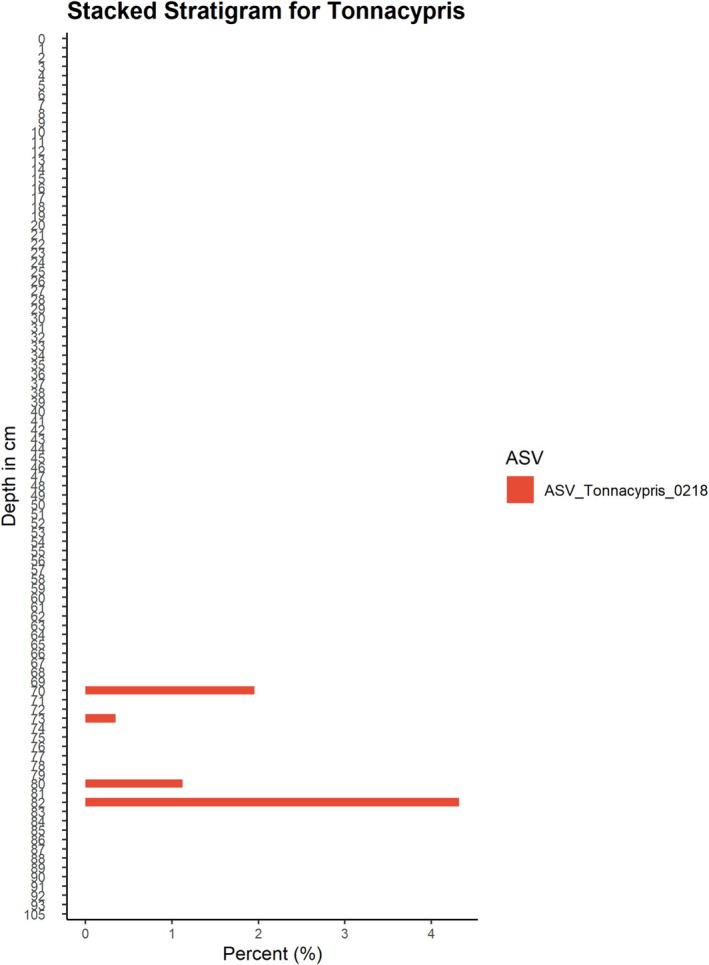
Stratigram showing stacked proportions of ASVs matched to Tonnacypris.

To the best of our knowledge, this is the first study investigating paleogenetic metabarcoding data of ostracods from high‐altitudinal areas. We reconstructed the genetic species assemblages during the last two millennia showing the connection between assemblage shifts and temperature and precipitation changes in Nam Co and its catchment. Furthermore, the data presented here indicated small proportions of intra‐species variation (see Appendix Figures E1, E2, E3, E4, E5, E6), especially during climatic transition phases. Despite the importance of genetic research, few studies have focused on the molecular ecology of non‐marine ostracods in high‐altitude lakes. Additionally, the taxonomic assessment of ostracods in remote regions, such as the Tibetan Plateau, and large lakes with potentially multiple endemic species needs more attention in the future to enable genetic research, as the here presented work. Excellent taxonomic knowledge and that of ecological preferences are absolutely necessary to apply paleogenetic analysis. Future research should prioritize whole‐genome sequencing of endemic ostracod species in Nam Co, comparative phylogeographic studies across the Tibetan Plateau, and investigations into the functional genomics of traits that enable survival in extreme environments combined with direct taxonomic classification and description of the morphological specimens. Understanding the genetic basis of tolerance to high UV radiation, low oxygen availability, and fluctuating salinity could provide broader ecological and evolutionary insights into how organisms adapt to extreme habitats. This would also offer valuable insights into the complex evolutionary history of non‐marine ostracod species.

## Author Contributions

**Katharina Dulias:** conceptualization (lead), formal analysis (lead), validation (lead), visualization (lead), writing – original draft (lead), writing – review and editing (lead). **Bernd Wünnemann:** data curation (equal), writing – review and editing (supporting). **Keshao Liu:** data curation (equal). **Yongqin Liu:** data curation (equal). **Junbo Wang:** writing – review and editing (supporting). **Antje Schwalb:** funding acquisition (lead), writing – review and editing (supporting). **Anja Schwarz:** formal analysis (equal), visualization (equal), writing – review and editing (equal). **Luis Victoria Nogales:** formal analysis (equal), methodology (equal), writing – original draft (supporting), writing – review and editing (equal). **Sten Anslan:** data curation (equal), investigation (equal), resources (equal), writing – review and editing (equal). **Wengang Kang:** writing – review and editing (supporting). **Ping Peng:** data curation (equal). **Nicole Börner:** writing – review and editing (supporting). **Paula Echeverría‐Galindo:** writing – review and editing (supporting).

## Funding

Funding was provided by the Deutsche Forschungsgemeinschaft (DFG) via the International Research Training Group “Geo‐ecosystems in transition on the Tibetan Plateau (TransTiP)”, DFG grant 317513741/GRK 2309.

## Conflicts of Interest

The authors declare no conflicts of interest.

## Data Availability

Raw sequencing data have been deposited in the Sequence Read Archive (SRA) under BioProject PRJNA781478 (accession numbers: SRR33514267–SRR33514365).

## Appendix A Age‐Depth Model From Anslan et al. (2022) and Covered Climate Periods

**TABLE A1:** Climate periods referenced in the text and figures.

Period name	Abbreviation	Start (CE)	End (CE)	Definition/Reference
Late Antique Cold Period	LACP	50	750	Cold period/Mischke and Zhang (2010)
Medieval Climate Anomaly	MCA	600	1400	Warm period, not homogenous across the TP/Li et al. (2022)
Little Ice Age	LIA	1350	1850	Cold event/Mischke and Zhang (2010)
Recent global warming	RGW	1950	Today	Warming/Liu and Chen (2000), Xie et al. (2010)

## Appendix B Constrained Cluster and Broken‐Stick Analysis

NOTE: Only reliably dated samples and their associated zones are depicted in the stratigrams of the main research article.

## Appendix C Diversity Metrics

To capture multiple characteristics of diversity, we combined several diversity indices. This is essential because no single metric captures all aspects of diversity. Richness reflects the number of species or variants, while indices like Simpson Evenness and the Simpson Index provide insights into relative abundances and dominance patterns. Turnover adds a temporal dimension, highlighting compositional changes over time. Faith's PD further adds the phylogenetic information. Together, these metrics offer a more comprehensive understanding of community structure, function, and dynamics, as well as broader ecological processes, enabling a better understanding of biodiversity distribution, drivers of change, and ecosystem resilience.

We selected the following diversity indices to measure temporal alpha‐ and beta‐diversity across the ostracod and diatom data:

*Richness*: Measures the number of different species or genetic variants in a community. Species richness is simply a count of species and does not take into account the abundances of the species or their relative abundance distributions.

*Simpson Evenness*: Captures how evenly individuals are distributed across species and is derived from the Simpson Index. The value of Simpson's D ranges from 0 to 1, with 0 representing infinite diversity and 1 representing no diversity. So, the larger D, the lower the diversity. For this reason, Simpson's index is usually expressed as its inverse (1/D).

*Inverse Simpson Index*: The higher the value, the higher the diversity; positively correlated with the Shannon diversity index.

*Shannon Diversity Index*: The higher the value, the higher the diversity of taxa in a particular community.

*Turnover*: Reflects the rate of species variant replacement over time, providing insights into community stability or shifts. Turnover is the calculated proportion of species that differ between time points, summed appearances and disappearances relative to total species richness across both time periods. The value ranges from 0 to 1.

*Phylogenetic Diversity* (*PD*): The PD value is calculated using the phylogeny of the species composition. The PD is defined as equal to the sum of the lengths of all branches of a phylogenetic tree of all members of the dataset (Faith 1992).

**TABLE C1:** Overview of ostracod diversity indices.

Depth in cm	Years in BCE	Rich‐ness	Simpson Evenness	Inverse Simpson	Shannon	Turnover	Appearance	Disappearance	Phylogenetic Diversity (PD)
0	2019	4	0.309913617	1.23965447	0.381406	0.25	0.25	0	0.921
1	2010	3	0.431613084	1.294839252	0.400782	0.25	0	0.25	0.677
2	2002	4	0.332125416	1.328501664	0.510119	0	0	0	0.946
3	1993	4	0.352551222	1.410204889	0.510216	0.25	0.25	0	0.896
4	1985	3	0.480934713	1.442804138	0.544684	0.25	0	0.25	0.971
5	1976	4	0.333724272	1.33489709	0.464542	0.5	0.5	0	0.921
6	1971	2	0.892648923	1.785297845	0.631749	0	0	0	0.728
7	1965	2	0.559357416	1.118714833	0.216435	0	0	0	0.677
8	1960	2	0.576219757	1.152439515	0.256754	0	0	0	0.677
9	1955	2	0.67753977	1.35507954	0.431391	0.33	0	0.33	0.694
10	1949	3	0.43150265	1.294507949	0.392386	0.33	0.33	0	0.863
11	1926	2	0.509800692	1.019601384	0.054648	0.5	0.5	0	0.778
12	1903	1	1	1	0	0.5	0	0.5	0.694
13	1880	2	0.623813614	1.247627228	0.350098	0.5	0.5	0	0.702
14	1857	1	1	1	0	0	0	0	0.711
15	1834	1	1	1	0	0	0	0	0.660
16	1806	1	1	1	0	0.5	0	0.5	0.686
17	1778	2	0.965996482	1.931992963	0.675442	0.5	0.5	0	0.744
18	1750	1	1	1	0	0	0	0	0.660
19	1722	1	1	1	0	0.67	0	0.67	0.711
20	1694	3	0.886497145	2.659491435	1.026465	0.67	0.67	0	0.904
21	1678	1	1	1	0	0.5	0	0.5	0.736
22	1663	2	0.533031706	1.066063411	0.141659	0.33	0	0.33	0.702
23	1647	3	0.333782187	1.00134656	0.006031	0.67	0.67	0	0.863
24	1631	1	1	1	0	0.5	0	0.5	0.711
25	1615	2	0.855222817	1.710445634	0.605938	0	0	0	0.728
26	1600	2	0.713060693	1.426121387	0.475647	0	0	0	0.702
27	1584	2	0.889485797	1.778971594	0.62967	0	0	0	0.677
28	1569	2	0.977558348	1.955116697	0.681624	0	0	0	0.677
29	1554	2	0.624440225	1.248880451	0.351171	0	0	0	0.711
30	1539	2	0.696117188	1.392234377	0.455322	0	0	0	0.728
31	1522	2	0.989555125	1.97911025	0.68786	0	0	0	0.702
32	1506	2	0.633121199	1.266242399	0.365694	0	0	0	0.727
33	1489	2	0.995168523	1.990337046	0.690718	0	0	0	0.702
34	1473	2	0.725377915	1.45075583	0.489614	0	0	0	0.677
35	1456	2	0.879603683	1.759207365	0.623056	0	0	0	0.677
36	1436	2	0.999817292	1.999634583	0.693056	0.5	0.5	0	0.677
37	1416	1	1	1	0	0.5	0	0.5	0.694
38	1396	2	0.562838913	1.125677825	0.225161	0.33	0	0.33	0.871
39	1377	3	0.349967085	1.049901256	0.119861	0	0	0	0.896
40	1358	3	0.721445154	2.164335461	0.836205	0.33	0.33	0	0.863
41	1337	2	0.925196353	1.850392706	0.652158	0.33	0	0.33	0.694
42	1316	3	0.528534313	1.58560294	0.560833	0.5	0.25	0.25	1.006
43	1296	3	0.343718162	1.031154487	0.089207	0.33	0.33	0	0.904
44	1275	2	0.995879634	1.991759268	0.691077	0.33	0	0.33	0.744
45	1255	3	0.383582808	1.150748425	0.276787	0.66	0.66	0	0.863
46	1218	1	1	1	0	0.5	0	0.5	0.677
47	1181	2	0.586652645	1.173305289	0.279506	0	0	0	0.711
48	1143	2	0.762255786	1.524511572	0.527871	0	0	0	0.719
49	1106	2	0.72096094	1.44192188	0.48468	0.33	0	0.33	0.677
50	1070	3	0.644059062	1.932177186	0.713416	0.33	0.33	0	0.972
51	1009	2	0.806481434	1.612962867	0.567842	0	0	0	0.736
52	948	2	0.587790528	1.175581056	0.281899	0	0	0	0.702
53	886	2	0.663711781	1.327423562	0.412325	0.33	0	0.33	0.677
54	826	3	0.343705419	1.031116258	0.089554	0.6	0.2	0.4	0.863
55	765	4	0.448738841	1.794955362	0.721666	0.25	0.25	0	1.048
56	697	3	0.466633273	1.39989982	0.506624	0.25	0	0.25	0.921
57	628	4	0.502847991	2.011391965	0.918972	0.5	0.5	0	1.048
58	560	2	0.607180419	1.214360838	0.320293	0	0	0	0.905
59	492	2	0.625686426	1.251372852	0.353295	0.5	0.5	0	0.897
60	424	1	1	1	0	0	0	0	0.660
61	356	1	1	1	0	0	0	0	0.660
62	289	1	1	1	0	0	0	0	0.660
63	222	1	1	1	0	0.5	0	0.5	0.694
64	154	2	0.736307361	1.472614722	0.501483	0.5	0.5	0	0.677
65	86	1	1	1	0	0	0	0	0.711
66	24	1	1	1	0	0	0	0	0.660
67	−40	1	1	1	0	0.5	0	0.5	0.677
68	−104	2	0.571090171	1.142180343	0.244986	0.5	0.5	0	0.753
69	−167	1	1	1	0	0.5	0	0.5	0.677
70	−230	2	0.519912417	1.039824834	0.096209	0.5	0.5	0	0.861
71	NA	1	1	1	0	0.67	0	0.67	0.660
72	NA	3	0.411569091	1.234707273	0.401627864	0.5	0.25	0.25	0.896
73	NA	3	0.381233543	1.143700628	0.259822975	0.75	0.5	0.25	1.038
74	NA	2	0.846275791	1.692551583	0.599351834	0.5	0.5	0	0.711
75	NA	1	1	1	0	0.67	0	0.67	0.694
76	NA	3	0.558402129	1.675206386	0.728064908	0.33	0.33	0	0.973
77	NA	2	0.556998361	1.113996721	0.21039069	0.5	0.5	0	0.736
78	NA	1	1	1	0	0	0	0	0.728
79	NA	1	1	1	0	0.75	0	0.75	0.694
80	NA	4	0.303044794	1.212179177	0.381818851	0.5	0.5	0	0.962
81	NA	2	0.609626321	1.219252643	0.324843222	0.33	0	0.33	0.854
82	NA	3	0.409950291	1.229850874	0.396720371	0.33	0.33	0	0.963
83	NA	2	0.543647678	1.087295356	0.173932589	0.33	0	0.33	0.897
84	NA	3	0.434543337	1.303630011	0.441678086	0.33	0.33	0	0.922
85	NA	2	0.563301148	1.126602296	0.226303258	0	0	0	0.888
86	NA	2	0.880497628	1.760995257	0.623661498	0.33	0	0.33	0.940
87	NA	3	0.800159974	2.400479921	0.94790666	0.33	0.33	0	0.913
88	NA	2	0.886221899	1.772443797	0.627505011	0	0	0	0.797
89	NA	2	0.595433647	1.190867293	0.297550862	0	0	0	0.677
90	NA	2	0.503167867	1.006335734	0.021335587	0.5	0	0	0.677
91	NA	2	0.874680091	1.749360183	0.61969266	0	0.5	0	0.685
92	NA	1	1	1	0	0	0	0	0.720
93	NA	1	1	1	0	0.67	0	0.67	0.728
106	NA	3	0.343122525	1.029367575	0.078222742				0.863

**TABLE C2:** Means of diversity indices per biostratigraphic zone of the ostracod data.

Depth in cm	Zone	Richness	Simpson Evenness	Inverse Simpson	Shannon	Turnover
5–0 cm	V	3.66	0.37	1.34	0.47	0.25
10–6 cm	IV	2.2	0.63	1.34	0.39	0.13
24–11 cm	III	1.57	0.85	1.21	0.16	0.42
57–25 cm	II	2.30	0.71	1.52	0.48	0.21
70–58 cm	I	1.38	0.85	1.10	0.12	0.30

## Appendix D Phylogenetic Diversity in the Core Section Below 70 cm

Four different ASVs with contributions of 3.971%, 6.42%, 4.22%, and 9.01% to the ostracod assemblage of different sampling points in the unreliably dated core section below 70 cm appeared only between a core depth of 74–88 cm. Unfortunately, due to the sediment disturbance of our core C8, no interpretation based on age and related environmental variables is possible. However, it is noteworthy to highlight the genetic diversity change between this older part of the core and the dated younger part. Especially since the ASVs in the dated section that differ from the most dominant ASV never reach more than 2.36% of the overall assemblage, while mainly contributing less than 0.5% each to the assemblage. Therefore, it appears that the conditions in Nam Co changed in favor of the dominating ASV from 167 bce.
